# The Experiences of Tobacco Use among South-Western Taiwanese Adolescent Males

**DOI:** 10.3390/ijerph120910522

**Published:** 2015-08-28

**Authors:** Rei-Mei Hong, Su-Er Guo, Mei-Yen Chen

**Affiliations:** 1 Chronic Disease and Promotion Research Center, School of Nursing, Chang Gung University of Science and Technology, Chiayi 613, Taiwan; E-Mails: seguo@mail.cgust.edu.tw (S.-E.G.) meiyen@mail.cgust.edu.tw (M.-Y.C.); 2 Research Center for Industry of Human Ecology, Taoyuan City 33303, Taiwan

**Keywords:** smoking behaviors, adolescent boys, school settings

## Abstract

Most smokers start young. Initiation of cigarette smoking at an earlier age leads to more life-years for tobacco use, makes quitting harder, and increases the risk of serious health consequences. Despite these challenges, research focusing on smoking behavior among adolescent boys in Taiwan is rare. Although the Taiwanese government enacted the Tobacco Hazards Prevention Act in 2009, aimed at prevention and reducing the rate of smoking, the percentage of high school students who smoke has continued to increase. In 2006, 7.5% of adolescent boys engaged in smoking. By 2012 the rate had increased to 24.6%. This paper explores the experiences that contribute to adolescent Taiwanese boys making the decision to smoke. A phenomenological approach to inquiry was used as the philosophical foundations for this study with twelve adolescent boys who engaged in smoking behaviors. Data was gathered through two face-to-face semi-structured interviews and a focus group. Data analysis was performed using Colaizzi’s analysis method. Findings indicated that decisions to begin smoking were motivated by curiosity and as a means of establishing friendships while decisions to continue smoking were linked to the addictive nature of smoking and as a means of coping with stress and passing the time. The findings can be used to inform the prevention of tobacco use and to reduce the high smoking rates among adolescent boys.

## 1. Introduction

Smoking is one of a number of high risk factors for lung cancers and other health-related chronic diseases [1,2]. Adolescents in Taiwan are increasingly experimenting with tobacco, especially in rural areas. In 2000, the percentage of high school students in Taiwan who had experimented with tobacco use on more than one occasion was 46.6%, and by 2010, this figure had increased to 66.4% [3]. For adolescents, initial experimentation with tobacco use has the potential to lead to addictive behaviors which in turn will have a long-term impact on their health and quality of life [4,5]. Several risk behaviors are more likely to occur in adolescents who smoke cigarettes such as involvement in fights, the carrying of weapons, suicide attempts, engaging in high-risk sexual behavior, and the use of alcohol and other drugs [6].

Adolescence is a critical stage for physical and psychological development. It is a time when young people begin to assert themselves, challenge authority, exercise what they believe are their rights and do not like to be controlled by rules [7,8]. Smoking is one of the behaviors that teenagers have used to express their freedom with most boys in Taiwan beginning to smoke between the ages of 11 and 14 [6,7], and becoming regular smokers by the age of 18 [9]. 

In recent years, anti-smoking has become a world trend. The Taiwanese government figures amongst those who have taken a stand against tobacco smoking enacting the Tobacco Hazards Prevention Acts in 2009, which includes: restrictions on tobacco advertising and promotion from television and radio programs, inhibits tobacco use in schools, and prohibits the sale of cigarettes to adolescents. Despite these measures, however, the percentage of adolescents smoking continues to rise [3]. 

While there have been some qualitative studies undertaken concerning tobacco use in adults [10], there is a noticeable absence of such research about adolescent boys’ experience of tobacco use in South-Western Taiwan. Therefore, the purpose of this study was to explore the lived experience of Tobacco Use among South-Western Taiwanese Adolescent Males. 

## 2. Methodology (Theoretical Framework)

The research study presented here used phenomenology as its methodology. A phenomenon is defined as *some-thing or situation* that is of interest to the researcher that gives rise to a process of inquiry about which little is known ([11], p.1071). Heidegger suggests that phenomena are *the totality of what lies in the light of day or can be brought to the light* (cited in [12] p.706). From a philosophical standpoint, the term phenomenon refers to perceived events, situations or objects of concern which surface in the everyday world of human beings [13]. In this study, the phenomenon of interest to this researcher is the experience of tobacco use among adolescent boys in Taiwan.

“Phenomenology is a way of uncovering the essential nature or structure of phenomena…, it is the primary mode of communicating experiences of phenomena as they occur in everyday life” ([14], p. 113). Phenomenology is the methodology of choice for a study such as this which seeks to understand behaviors as representations of human everyday experiences that impact on the health and well-being of individuals. It offers a way of systematically studying and learning about phenomena that is typically difficult to observe or measure” ([15], p. 99).

The centrality of embodied human experience as a point of exploration of the lived world of people and objects is underpinned by two concepts—intentionality and the phenomenological attitude. The term intention as used in common language refers to a person’s agenda, mental state or plan of action to achieve a specified goal. It is a stance that a person takes with the intent of propelling one forward to a particular outcome. On the other hand the concept of intentionality within phenomenological thought posits that human beings are inextricably related to the context in which they live out their daily pattern of life [16]. The natural attitude pertains to the beliefs and theoretical conceptions held by humans about the ways in which human beings engage with the world of persons and objects as they go about their ordinary everyday life. The notion of ordinary everyday life is meant to denote the activity of living in which much of the person’s world is acted out through assumed and uncontested awareness [17].

The philosophical underpinnings of phenomenological thought are consistent with the values of human life, the uniqueness of the person, the importance of personal discovery, acceptance of life situations, the need for exploration of meaning, constructed reality and the potential for person growth. As a philosophy, phenomenology is concerned with the question of *knowing how human beings know and how knowledge is manifested to the human person* ([18], p.10). The exclusive focus on experience therefore, provides access to all that can be directly known, because all knowledge is ultimately grounded in experience [19]. In this context, phenomenology as a philosophical framework and research methodology can provide knowledge about aspects of a person’s life in health and illness which cannot be accessed by observation alone. 

## 3. Method

### 3.1. Participants and Ethics

Participants in this study were high school students aged between 13 and 18 years who self-identified as having smoked on more than two occasions, were current smokers, had experiences of attending smoking prevention programs, and willing to share their experiences of smoking. Ethics approval was granted from the Human Research Ethics Committee of the University Hospital. Once ethics approval had been obtained and permission from the President of the High School was received the researcher circulated a flyer inviting interested students to be part of the study. The school nurse explained the purpose to the students who would like to join the study. Acceptance of the invitation required the students to contact the researcher by phone to express a personal interest in being interviewed about their experiences of smoking. Prior to the commencement of the study, informed consent was obtained in writing from the participants and their guardian(s). 

In qualitative inquiry—phenomenology in this instance—the number of participants is generally small in comparison to quantitative research. The actual number of participants in qualitative inquiry is flexible, dependent on what is generally termed *data saturation*—the point at which repetition of information occurs with no evidence of new knowledge occurring [20,21,22,23]. Participant numbers are secondary to the importance of obtaining the richest possible sources of information to answer the research question. In terms of recommended numbers, respected suggestions have been put forward by Morse [24] and Parse [25] who purport that the actual number of participants can range from three to ten depending on the type of qualitative study being undertaken and at what point the quality of redundancy is achieved. Redundancy, or data saturation occurs when the researcher becomes aware of a pattern that is repeated in the descriptions of the participants. The initial intent of this researcher was to interview between eight and 10 participants in keeping with the counsel of Parse [25]; however, data saturation was not achieved until participant 12. 

### 3.2. Information Gathering

Information was gathered through in-depth focused interviews in Taiwanese. Each participant was interviewed twice. The purpose of the first interview was to explore participants’ experiences of smoking while the purpose of the second was to provide an opportunity for the participants to review their respective transcript of data and give more explanation. The first interview ranged from 40 to 60 min while the second interview ranged from 30–40 min. One focus group interview was conducted after the second interviews in order to gather richer data by asking participants to produce a drawing and share their ideas about their experience of smoking. All interviews and the one focus group were conducted in the school’s consulting room. Twelve participants attended. Throughout the interview process the participants were provided with both time and space to explore their world of experience within an atmosphere of personal respect and humility. All interviews were audio-recorded. During the interviews the researcher took notes for further reference during the analysis process. Research questions to encourage the participants to elaborate on their experiences were recursive in nature: questions leading to clarification of participants’ experiences aimed at eliciting as full a description of the phenomenon as possible. Examples of such recursive questions are presented in Table 1. At the completion of each interview the researcher set aside time for quiet reflection on the interview process after which she made notes for further reference during the analysis process.

**Table 1:** The flow of the topics used during the interviews.

Participants	Interview Questions
Participant interviews (first round) (n = 12)	Introduce yourself Tell me about your family background. Initiation of cigarette smoking? Tell me more about your thoughts and feelings related to your experiences of smoking, Tell me more about the stories.
Participant interviews (second round) (n = 12)	Can you give an example of your experience? Can you describe your image of smoking? Tell me more about your feeling?
Focus group (n = 12)	How do you think about yourself? Could you draw what you think when you are smoking? Share with others about your experiences of smoking Share woth others about what you think about yourself.

The participants’ identity was protected by the use of pseudonyms. As it is a common practice among youth in Taiwan to have an English name, the participants were asked to choose an English name as their pseudonym. 

### 3.3. Data Analysis

The process of analysis used Colaizzi’s [26] method which involved the following steps. The transcriptions of interviews were read and re-read to gain an overall understanding of each participant’s experience of the phenomenon. Once the researcher felt comfortable that she had gained a general understanding of the participants’ experiences, a review of each transcript was undertaken to identify significant statements (statements that directly related to the phenomenon). The participant statements were then formulated into meaning units which were then aggregated into theme clusters significant statements and formulated meaning units were reviewed by the researchers and the participants to ensure that the analysis process reflected the participants’ experiences [27]. 

The place of validity and reliability in phenomenological research has been the source of considerable debate in the scientific community [28,29]. This debate is generated by virtue of differences in paradigm adherence, selected methods and, theoretical considerations. Rigour in phenomenological inquiry is demonstrated through the researcher’s attention to and transparency of the conduct of the researcher throughout the course of the study [30]. There are a number of differing frameworks for ensuring rigour in qualitative research [31,32]. Streubert and Carpenter have developed four constructs for ensuring rigour of a study. These constructs are credibility, transferability, dependability and confirmability [30]. In order to achieve credibility in this study this researcher returned the findings to participants to ensure accuracy of interpretation. Moreover, the research described as faithfully as possible each part of the research process employed in this study. In terms of this study, at the completion of this inquiry the findings were made available to other groups of adolescent boys who smoked for their own personal reflection. Dependability is concerned with the stability of the information over time with a need to be able to demonstrate any changes, shifts or deviations from the original text. A continuing audit of the research process was undertaken by the researcher and her two co-authors. Confirmability is established when credibility, transferability and dependability are achieved [33]. This researcher ensured that the processes involved in conducting this study were systematically documented for the purpose of transparency. 

## 4. Findings

The process of analysis using Colaizzi’s analysis revealed five themes: (1) motivated by curiosity; (2) a means of establishing friendships; (3) unable to beat the habit; (4) a means of coping with stress; and (5) a means of passing time. 

### 4.1. Theme One: Motivated by Curiosity

All participants described experiences of feeling curious or excited when they saw tobacco. They stated that they were curious about the taste and so wanted to try tobacco.

- Michael said: “I started smoking because I wanted to find out what it would be like.
- John also had this experience of smoking: “My mother and father both smoked. I wanted to try it when my friend gave it to me.”
- Damien further indicated: “I felt excited and curious about smoking. I first tried tobacco when I was 12 years-old. At that time watching the men in movies smoking seemed powerful and cool. I wanted to be like them.”
- Steve stated: “I was talking with my friends and they were smoking. My friend gave me my first cigarette. At that moment, I just wanted to try it. The first time I smoked it felt painful. However, later, I felt relaxed.”
- Wayne’s comments also supported this, as he stated: “I sometimes cannot stand not to smoke and then I smoke in the classroom. So the school knows my secret, and they made a note of this on my school record. I was curious about cigarette which is what made me smoke my first one. I remember that I was in grade six at that time. All of my family smoked. I did not think that it was a bad habit.”

Curiosity is a factor linked to male adolescent smoking. One research study that examined male adolescents in China found above half of the participants had experiences of smoking, and described the motivation of beginning smoking as mainly curiosity [34]. 

### 4.2. Theme Two: A Means of Establishing Friendships 

The role of friends as an influencing factor featured in all 12 participants interviews. These friends were all people they had either grown up with or who were neighbors. The participants all stated that one of the major influences on their decision to smoke was their desire not to destroy their relationship with their friends. Through smoking they found a niche in which to build and maintain their self-confidence and support network. With support from friends, they felt that life was meaningful and smoking acted as a conduit for this.

- John said: “my senior-high school friend gave it [tobacco] to me. He was my role model. We grew up together.
- For Peter, smoking was a way to overcome a lack of belief in himself: “I did not want to reject my friend. Friendship was important to me. Almost all of my friends smoked. Friends are all I have. Without them, I did not know what to do.”
- Steven indicated that: “my friend gave me the first cigarette when I was at elementary school [grade six]. I wanted to make friends, otherwise, I would be so lonely at the school. Friends really mean a lot to me in my life and my best friends all smoke.”
- John also pointed out that: “The first time I smoked was outside of my home. My friends gave it [the cigarette] to me. I didn’t feel at all uncomfortable. If my friends had not given me the cigarette then I would not smoke. These friends are all people that I know from outside of the school. Some of us grew up together.”
- Roger also indicated that friends were central to his decision: “my friends wanted me to smoke, so I smoked, I could not reject it. Otherwise, I would lose the friendship. I felt bored, and I needed friends.”

Smoking in order to establish friendship was also considered by Naing, Ahmad, Musa, Hamid, Ghazail, and Bakar [5] to be one of the factors related to smoking habits of male adolescents. Nargiso, Becker, Wolff, Uhl, Simon, Spirito, and Prinstein [35] also found that friends’ cigarette use had a great effect on smoking outcomes within adolescents. Peer influence was generally the major reason for them taking the habit. Having close friends who smoked, and having close friends who encouraged boys to smoke were the risk factors associated with their occasional and regular smoking [36,37]. 

### 4.3. Theme Three: Unable to Beat the Habit

For a number of the participants, their tobacco use had become a habit. There was a significant association between smoking and enjoyment and relaxation in free time among male adolescents [39].

- Steven said: “I smoked during the time from upon waking, and after the meal at night Smoking has become my daily habit. I usually smoked twice per day. Generally, I smoke five cigarettes per day. After dinner, I want to smoke. Maybe it was because my father smoked as well. I followed his habit. However, I really could not stop smoking when my father and my grandfather smoked at that moment.”
- David also had the same opinion: “When I wake up, I want to smoke. It is my hobby. My family all smoke after meals. It is how my family lives their everyday life.”
- John further demonstrated the normalcy of smoking: “I smoke when my father picks me up from school. My father gives me the cigarette without any concern, and I just grab it and smoke it without anyone noticing. Smoking has become my habit.”
- John further demonstrated his identity as a habitual smoker by producing a drawing of himself smoking. I just smoke after meals or during chatting with my friends. Smoking became our habit when my friends and I talked and when I made friends.
- Peter further said: “I smoke five or six [cigarettes] per day. It has become my habit. I usually save my breakfast money to buy cigarettes. My family all smoke. My parents know I smoke. If my parents chastised me, I would smoke less. But they don’t.”
- Frank even said I smoked when I saw cigarette at the dinner table. It must be left by my father. I did not know why he left it there; however, I would like to smoke when I saw the cigarette. I used to grab one cigarette and smoke. I could not stand it.

The inability to stop smoking among adolescent boys is strongly associated with their fathers smoking [5]. Male high students were found that of the male high school students with smoking experience, half of them bought cigarette from vending machines. They thought of smoking when they saw cigarettes [37]. 

### 4.4. Theme Four: A Means of Coping with Stress 

A number of the participants linked their decision to continue smoking to their experience of smoking as a way to relieve or cope with stress. Peter found that smoking made his “bad mood to go away” and that his academic assignments and stress put him under a lot of stress that caused him to smoke.”

- Wayne described a similar situation: “When I had bad mood, I smoked. My parents both work hard and nobody takes care of me. At the school, my classmates did not like me. I do not know what to do. I just go from day to day without any plan or dream.”

The feeling of disempowerment for these adolescent boys with no ability to make personal choices for their goals in the future is strong and very influential on their decisions around smoking.

- John drew himself: “I was just like an ugly vampire. I felt stressed most of the time. The academic assignments and classes made me stressed. I was under pressure. Smoking had helped me feel less stressed, but I also now eat more. Most of my friends have smoked since grade six. Their parents are all busy at work, and have no time to care for them.”

Karpinski, Timpe and Lubsch [38] reviewed several articles and found that male adolescents smoked when they had a bad mood or when they were under stress.” For the boys, smoking helped them to cope with bad moods, worries, and depression. 

### 4.5. Theme Five: A Means of Passing the Time 

The lifestyle in the South-Western counties of Taiwan is seen, by the participants, as boring for adolescent youth. Steven voiced his concerns about leisure and the need to relax after the stress of heavy academic burden with smoking becoming his way of “passing the time”. Each attempt to engage him in conversation about what was happening in his life usually ended in the response, “bored.”

The dilemma of not knowing what to do instead of smoking was at times untenable. However, in regards to smoking becoming a hobby to pass the time, the lines of communication opened up.” John comes from a wealthy family in Taipei. His family moved to the South-Western when he was in junior high school. John links his relocation to the South-West with his smoking: “I never smoked when I was in Taipei. After moving to here, I felt bored so I smoked. I decided to try to make friends by giving them cigarettes, and I found it worked. I have a lot of friends.”

- Steven also drew himself when he was feeling bored. “When I feel bored, I think of smoking. I feel happy and excited. Maybe only drawing can help me stopping smoking.”
- George confirmed the link between boredom and smoking stating: “I do not have anything to do, so I smoke. My parents are at work the whole day. They leave home early, and come home late. Sometimes, they work at night. Nobody talks to me. My sister and brother both live away from home. I feel bored.”

For most participants, they started smoking because of feelings of boredom and frustration. All participants did not like studying; and their academic performances were not good as well. They did not want to continue studying after high school graduation. Most participants had part-time jobs. They all spoke to the researchers: Making money is more important than education. Therefore, the government and health care professionals need to work hard not only on anti-smoking strategies like the enacted Tobacco Hazards Prevention Act, but also on creating some activities or skills or sports for this group. 

Wayne and Steven also pointed that “we sometimes smoked at school when we felt nothing to do. We did not like studying, especially in mathematics. The academic class made me dozing in the classroom. Only smoking can make me feel excited. I like painting cartoon. Maybe painting cartoon can help me stop smoking.” 

A central feature from this analysis is that all the themes are in some ways linked and as such interdependent. For the participants of this study, smoking is a habit, it kills time when they were bored and have nothing to do. For some adolescent boys, smoking seemed to relieve boredom [16]. For most participants, their parents worked and they felt that they had little time to be interested in what they did, especially when it came to smoking as for many it was a normal family practice which did not seem to warrant sanctions. Peer pressure and the need to belong also meant that these adolescent boys started to make friends with outsiders, and began smoking. The five themes presented above are discussed in relation to available literature on the topic to establish their significance for knowledge enhancement, family practice and future research on the experiences of smoking behavior among adolescent boys.

## 5. Discussion

According to the participants, their first cigarette generally came from their friends (especially close friends). They usually made friends by smoking and gathered together to smoke. Most of their parents or grandparents smoke. It was difficult for adolescents to stop smoking because their close friends smoked. Friends are the most important factors for them to control their smoking behavior [39]. Thrul *et al.* [39] also found that adult smoking in the community significantly influenced adolescents’ smoking behaviors. This study also indicated that parents were the most significant role models in adolescents’ life. Most male adolescents described that they would like to smoke less or quit; however, they saw their parents smoking which influenced their decisions. Parents who affected adolescents' smoking status could be involved for future prevention program. 

Most of the adolescent boys who smoked said that they smoked because they felt stress, especially from school work and parents’ pressure. Stress made them smoke. They thought that the idea about “to be a scholar was to be the top of society” was out of date, it should be “every profession produces its own best” instead. Taiwanese parents and society need to open their minds to accept or respect different points of view in terms of success for children. A successful life for adolescence is not necessarily academic achievement at school, but is a positive and thoughtful attitude or active involvement. 

Most smokers start young. Many school-based smoking prevention programs start at senior high schools; however, the researchers find that they need more time and strategies to control their smoking behaviors [40]. Although smoking is found mostly among adolescent boys, a school-based program in the elementary schools would be preferred to reach pre-teen boys. This study provides new insights and understanding into the life-world of adolescent boys with smoking experiences in Taiwan. The experience of tobacco use among adolescent boys in Taiwan contributes to the advancement of knowledge from a trans-cultural perspective. For most smoking adolescent male, smoking is a key for making friends, becoming a habit and coping stress. Wilson, *et al.* [41] reviewed 84 articles related to tobacco control and found increasing tobacco prices, moderating smoking bans and antismoking media campaigns were effective for controlling smoking behaviors among adolescents. Further studies could be undertaken involving government, media, community health professionals, school teachers and family members concerning the impact on their respective lives. 

## 6. Conclusions

The experience of tobacco use among adolescent boys encompassed a range of experiences involving: (1) motivated by curiosity; (2) a means of establishing friendships; (3) unable to beat the habit; (4) a means of coping with stress; and (5) a means of passing time. The findings of this study have the potential to make a significant contribution to extant knowledge for nurses who are caring for adolescent boys who smoke. Special attention should be given to those adolescent male smokers, and their parents who have mental health needs. Moreover, the development of a directory or web-site which provides useful resources for adolescent male smokers and their parents is necessary. Finally, policy initiatives in the areas of mental health intervention are required at a regional and national level. 

### 6.1. Limitations of This Study

The limitations involved in the conduct of this study are as follows:

1. The small number of participants from one region of Taiwan excludes generalisation of findings (although, this is not the intent of a qualitative study).
2. Conducting a study in two languages has the potential to create difficulties in accuracy of translation and ensuring the essential meaning of what is shared by participants is not lost or distorted. Difficulties were encountered by the researcher as a result of having to translate participant interviews. However, every means was taken to ensure that the integrity of the information gathering process and the process of analysis was not compromised.
3. Confirmation of findings by all 12 participants was not achieved as three participants declined the offer to review their transcripts of interviews.

### 6.2. Implications 

The findings of this study provided new insights and understandings of what it means to be an adolescent male smoker in Taiwan. The findings of this study highlight significant motivational factors that have lead these participants to engage in smoking behavior. Understanding the motivation for such behaviors is fundamental to the development of successful intervention programs and meaningful health promotion strategies. The findings also has the potential to provide direction for government policy initiatives in this area of research. 

### 6.3. Recommendations

1. Specific attention should be given to the mental health needs of youth smokers and their parents. This could take the form of in-service education or as units within post-graduate nurse education programs.
2. Establish a referral and community support network between government and non-government organizations involved in providing support for adolescent male smokers and their parents.
3. Establish a support infrastructure within the school system to address the psycho-social and educational needs of these youth smokers.
4. Policy development reform be undertaken to focus on the needs of disadvantaged groups, in this instance, youth smokers and their parents especially in relation to social welfare payments, housing, and access to the workforce.
5. Future research to be undertaken concerning mental health concerns for youth smokers and their parents in Taiwan.
